# A Wandering Mind Does Not Stray Far from Home: The Value of Metacognition in Distant Search

**DOI:** 10.1371/journal.pone.0126865

**Published:** 2015-05-14

**Authors:** Ravi S. Kudesia, Markus Baer, Hillary Anger Elfenbein

**Affiliations:** Department of Organizational Behavior, Olin Business School, Washington University in St. Louis, St. Louis, Missouri, United States of America; Canterbury Christ Church University, UNITED KINGDOM

## Abstract

When faced with a problem, how do individuals search for potential solutions? In this article, we explore the cognitive processes that lead to local search (i.e., identifying options closest to existing solutions) and distant search (i.e., identifying options of a qualitatively different nature than existing solutions). We suggest that mind wandering is likely to lead to local search because it operates by spreading activation from initial ideas to closely associated ideas. This reduces the likelihood of accessing a qualitatively different solution. However, instead of getting lost in thought, individuals can also step back and monitor their thoughts from a detached perspective. Such mindful metacognition, we suggest, is likely to lead to distant search because it redistributes activation away from initial ideas to other, less strongly associated, ideas. This hypothesis was confirmed across two studies. Thus, getting lost in thoughts is helpful when one is on the right track and needs only a local search whereas stepping back from thoughts is helpful when one needs distant search to produce a change in perspective.

## Introduction

**Fig 1:**
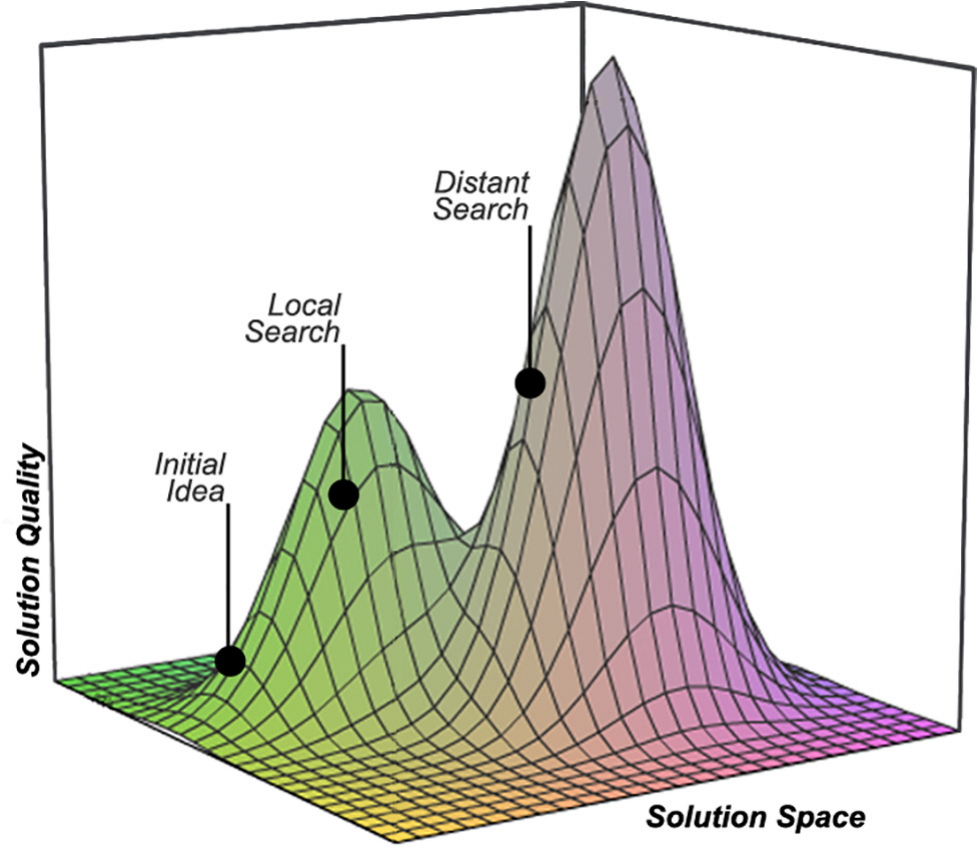
Local and Distant Search. Search processes within a hypothetical solution space.

Understanding how humans solve problems is, paradoxically, a problem in itself. The earliest models from game theory identified axioms that rational agents should follow to decide among various solutions to their problems [1]. However, this focus on deciding *among* solutions left open the question of how agents *identified* solutions in the first place. Realizing that people could not possibly consider all the potential solutions contained in their memory, much less solutions of which they have no knowledge, behavioral models have highlighted the *search* process [2,3]. How do agents search for solutions to their problems? In addressing this question, we can visualize the space of potential solutions as a landscape with peaks and valleys—as shown in Fig 1 (see [4]). The vertical dimension represents solution quality such that higher quality solutions are located higher up on the landscape’s peaks. Agents begin at an initial idea: their current solution or the first solution that comes to mind. Search can proceed from this initial idea in one of two ways [5]. If agents search for small incremental improvements on their initial idea, they can climb higher until they reach the best possible version of this particular solution. This incremental improvement process is known as *local search*, as agents explore only solutions that are close neighbors to their initial idea. While local search improves upon the initial idea, it does so in a limited way. Local search reflects the kind of thought process that put Polaroid into bankruptcy. Its managers kept locally improving its analog business instead of searching for the distant solution to their financial woes: digital photography [6]. In contrast to local search, *distant search* explores solutions that are fundamentally different in nature from one’s initial ideas [5]. In the solution space shown in Fig 1, the agent is better off engaging in distant search away from his or her initial idea than incrementally improving the initial idea through local search, though this is not always the case.

In this article, we explore the cognitive processes underlying local and distant search. While not differentiating local from distant search, extant work shows that letting one’s mind wander can help agents identify new potential solutions, whereas focusing attention does not [7]. Mind wandering refers to allowing thoughts to flow freely, whereas focused attention only allows specific types of thoughts to emerge and constrains all others. We suggest that letting the mind wander is likely to facilitate local search, but not distant search. We do so because mind wandering engages associational processes in which activation spreads from one’s initial idea to the ideas most strongly associated with it [8,9]. This will facilitate incremental local improvements, as these newly activated ideas are conceptually similar to the initial idea. Focusing attention, however, does not engage these associative processes, which is why it produces little benefit in identifying new solutions [7]. Given that activation in memory is path dependent, and thus heavily influenced by initial ideas, how then can distant search be possible? The predominant explanation is that when local search starts producing diminishing marginal returns, activation may be removed from unhelpful associates of the initial idea and redistributed to other ideas that have not been previously considered [10,11]. With enough redistribution of activation, one may suddenly see the problem in an entirely new light, enabling distant search. We suggest another process that could enable distant search without first requiring failure of local search. An emerging body of work suggests that agents can self-induce redistribution of activation through mindful metacognition [12–14]. This process, first identified by Buddhist meditation, runs directly counter to mind wandering [15]. Instead of being immersed in thoughts and getting lost in them, it describes how individuals can mentally “step back” and monitor their thoughts from an inner distance. Doing so reduces activation from these thoughts and redistributes it to others [12]. It is therefore frequently used in therapy settings as it allows patients to step back from their initial ideas about how to handle a situation, which are often maladaptive, and realize that there are other, more adaptive, solutions to their problems [16]. By redistributing activation away from one’s initial thoughts about a situation, agents can consider a wider range of possible solutions the next time they encounter the situation [12–14]. This wider range of solutions is possible because they approach the situation from a mindful state: they attend to the situation more fully, without the filter of habitual thoughts from the past [15]. We therefore suggest that mindful metacognition may facilitate distant search as it redistributes activation away from initial ideas towards other, less conceptually similar, ideas.

## Overview of Studies

We tested our argument—that mind wandering induces local search whereas mindful metacognition induces distant search—across two studies by adapting a paradigm from creativity research [17,18]. In it, participants worked on solving problems, then engaged in an experimental task designed to induce mindful metacognition, mind wandering, or a focused attention control, and then returned to problem solving. We assessed whether the task produced local or distant search by quantifying the degree of change in content from their initial ideas to their final ideas. Both studies shared this paradigm and differed only in the number and type of problems for which we assessed search. We therefore outline the shared paradigm in greater detail below.

Participants entered the laboratory—typically in groups of 2 to 6—and were seated at individual cubicles. On their computers, they then read and agreed to a statement of informed consent before proceeding to the experiment. Once the experiment began, participants worked on problem solving for two minutes per problem and reported their initial ideas. Then, they engaged in a randomly assigned ten-minute task that experimentally induced mind wandering, mindful metacognition, or a focused attention control condition. All three conditions were designed based on previous research [7,18–20]. As low-effort and simplistic tasks reliably induce mind wandering [9,21], participants in the mind wandering condition listened to audio describing the history of an English village and were required merely to press a button every 60 seconds as indicated by an on-screen counter. In the mindful metacognition condition, participants listened to audio narrated by the same individual as in the mind wandering condition, except this audio asked them to step back and monitor their thoughts from an inner distance, as if their thoughts were merely clouds passing in the sky [15,19,20]. As tasks requiring focused attention produce little benefit for search [7,18], our control task required participants to listen to the same history audio as the mind wandering condition, but had to focus on the audio and push a button each time it mentioned a specific person, place, or thing.

After the ten minutes of audio ended, participants reported their current levels of affect and the degree to which they were in a mindful state. They did so using single item measures from previous research [20]. We theorized that mindful metacognition would improve distant search by inducing a mindful state in which participants bring a more thorough and less habituated attention to the present moment. However, as mindful metacognition could also improve participants’ mood, we measured state affect as a potential confound. We assessed state mindfulness on an 11-pt Likert scale (“At this moment (right now), I feel like I will rush through activities without paying attention to them”). The item, derived from a common mindfulness scale [22], was reverse-coded such that higher agreement represented a less mindful state. We measured state affect using the Self-Assessment Manikin, which uses images on a 9-pt Likert scale to assess whether participants are in a negative or positive mood [23].

Participants then returned to the same problems they worked on prior to the break. Consistent with prior research (e.g., [7]), they did not expect to see these problems again. As before, they were given two minutes to solve each problem. After finishing this final problem solving section, they provided demographic information. We also included a debriefing question to address a potential confound: participants in certain conditions may have worked on the problems during their experimental tasks. The debriefing question (“Before you performed the audio-task, you were asked to solve some cognitive challenges. How often did you think about the problems during the audio-task?”) was measured on a 7-pt Likert scale. A one-way ANOVA on this question revealed no significant differences across conditions, *F*(2,210) = 0.24, *p* = .788, ω^2^ = 0.00, thereby ruling out this potential confound. Once all participants had finished, the experimenter concluded by explaining the purpose of this study, responding to any questions, and thanking participants for their time. Both studies took no longer than 30 minutes and were approved by the Institutional Review Board at Washington University in St. Louis.

## Study 1

### Participants

102 undergraduate students (55% female; 19.4 mean age) completed the study for course credit.

### Problem Type

Participants engaged in a divergent thinking paradigm that asked them to list as many unusual uses for a brick as they could within two minutes [24]. Divergent thinking problems are ideal to assess the content of search because there is no single correct answer. As such, participants produce a number of responses that allows us to track changes in content over time and across conditions. For example, if a participant’s initial idea envisioned using the brick as a tool to hammer nails, local search would show subsequent tool-related uses (e.g., sanding uneven surfaces). Distant search, however, would represent uses in a qualitatively different category, perhaps related to art (e.g., breaking the brick apart to produce pigment for painting). Therefore, our first hypothesis predicts that mind wandering and mindful metacognition will both lead to the identification of new uses (i.e., general improvement in search) with respect to the control condition. Our second hypothesis is that only mindful metacognition will lead to identification of new uses in qualitatively different categories from their initial ideas (i.e., distant search).

### Results and Discussion

All uses for a brick aside from its usual use—to build a structure—were counted as unusual. To test whether search improved as the result of our conditions, two condition-blind raters counted the number of unusual uses reported by participants after the experimental induction that were not direct repeats from their initial attempts (*r* = .95). This allowed us to test whether any differences existed across conditions in the number of new uses identified after the induction. A one-way ANOVA revealed significant differences across conditions, *F*(2,99) = 7.97, *p*<.01, ω^2^ = 0.12. In support of Hypothesis 1, a planned contrast showed that mind wandering (*M* = 4.03, *SD* = 2.37) and mindful metacognition (*M* = 4.35, *SD* = 2.20) improved general search relative to the control condition, (*M* = 2.26, *SD* = 2.39), *t*(99) = 3.95, *p*<.001, *d* = .82. These results suggest that mind wandering and mindful metacognition improve search in general, but do not speak to whether they reflect different types of search (i.e., local versus distant).

Therefore, we next tested Hypothesis 2, namely that only mindful metacognition would lead to distant search. To do so, in line with past work (e.g., [25]), the first author developed a list of categories based on common participant responses (using the brick as a tool, as art, etc.). The list showed a high degree of consistency with an independently developed list [25] and is available upon request. Two condition-blind raters then classified all solutions into these categories (*κ*_*c*_ = .84). Next, we counted the number of post-induction solutions belonging to a category the participant had not utilized before the induction. This measure directly assesses whether participants demonstrated a qualitative shift in the type of solutions they identified (i.e., distant search), as opposed to producing more solutions within the categories they had initially identified (i.e., local search). Given heterogeneous variances across conditions for this measure of distant search, we ran a one-way ANOVA using Welch’s adjusted F-ratio. It revealed significant differences in distant search across conditions, *F*(2,64.63) = 4.66, *p*<.05, ω^2^ = 0.07. In support of Hypothesis 2, a planned contrast showed that participants in the mindful metacognition condition (*M* = 2.74, *SD* = 2.08) identified more new categories than those in the mind wandering (*M* = 1.85, *SD* = 1.44) and control (*M* = 1.32, *SD* = 1.70) conditions, *t*(52.44) = 2.84, *p*<.01, *d* = 0.83. These results support our prediction that only mindful metacognition would enable distant search into ideas qualitatively different from participants’ initial ideas.

We further suggested that mindful metacognition operates by inducing a mindful state and not by improving affect, a possible confound. We therefore ran a multiple mediation model that included both state mindfulness and state affect [26]. The relation between the mindful metacognition condition and distant search was reduced to non-significance (*β* = .62, *p*<.01 to *β* = .28, *p* = .23) when controlling for state mindfulness and state affect. A bootstrap analysis showed that the 95% bias-corrected confidence interval for the indirect effect of state mindfulness excluded zero [.04,. 62], while that of state affect [-.10,. 22] did not. This suggests that the mindful metacognition condition operates by inducing a mindful state in participants, not by improving their mood.

Taken as a whole, this study supports our argument that mind wandering produces local search whereas mindful metacognition produces distant search. It does so because although both conditions produced new solutions (i.e., search in general), only mindful metacognition produced solutions that were qualitatively different from participants’ initial ideas (i.e., distant search).

## Study 2

Whereas the prior study’s divergent thinking paradigm allowed for an assessment of the content of search because it allowed for many correct solutions, this study used a convergent thinking paradigm in which there is a single right answer [24].

### Participants

111 undergraduate students (44% female; 19.5 mean age) completed the study for course credit.

### Problem Type

We used two insight problems, which cannot be solved by local search because they are specifically designed to trigger initial ideas that are incorrect [10,11]. The first problem asks, “Is it legal for a man to marry his widow’s sister?” In this case, initial ideas typically focus on legality. The solution cannot be reached by local search of ideas associated with legality. Solution requires participants to bypass legality and realize that a man with a widow is already deceased and cannot marry. The second problem asks, “Marsha and Marjorie were born on the same day of the same month of the same year to the same mother and the same father, yet they are not twins. How is that possible?” Initial ideas fixate on the idea of possibility, typically producing unique responses related to adoption or in vitro fertilization. Solution requires one to bypass activation of these unique possibilities and instead recognize that Marsha and Marjorie can be triplets. As insight problems are designed to trigger incorrect initial ideas, we hypothesized that only mindful metacognition would lead to solution because they require distant search—not local search. Participants had two minutes to solve each problem. We chose to ask two insight problems because each additional problem asked interferes with search processes of the previous problem, creating a diminishing marginal return [27]. We did not use only one problem because a large number of participants may be excluded if we only utilized one problem and they got that problem correct.

### Results and Discussion

Correct responses were scored dichotomously by two condition-blind raters (*r* = .97). Thirteen participants were excluded from analyses because they received perfect pre-induction scores, eliminating the need for search. For the remaining 98, distant search was measured as the improvement in correct responses from pre-induction to post-induction. As many participants showed no improvement, we analyzed these distant search scores nonparametrically to account for potential floor effects. A significant Kruskal-Wallis H test revealed differences between conditions, χ²(2) = 6.59, *p*<.05, η^2^ = 0.07. In support of our hypothesis, a planned contrast using the Mann-Whitney U test showed that distant search was higher for participants in the mindful metacognition condition (*M* = .28, *SD* = .43) than for those in the mind wandering (*M* = .06, *SD* = .42) and control (*M* = .08, *SD* = .26) conditions, *z* = 2.51, *p*<.05, *d* = 0.52.

As with the previous study, we ran a bootstrapped multiple mediation model to test whether distant search was driven by a mindful state or state affect. Because bootstrapping makes no distributional assumptions, this mediation analysis is not impacted by potential floor effects [26]. The relation between the mindful metacognition condition and distant search became non-significant (*β* = .54, *p*<.05 to *β* = .40, *p* = .09) after introducing state mindfulness and state affect as mediators. As predicted, the 95% bias-corrected confidence interval of the indirect effect of state mindfulness excluded zero [.09,. 56], while that of state affect [-.43,. 04] did not, suggesting that the mindful metacognition condition worked through the proposed mechanism.

This study thus further supports our hypothesis that mindful metacognition produces distant search, as only this condition facilitated problem solving on insight problems, which by definition require distant search and cannot be solved by local search.

## Conclusions

In this article, we explored the cognitive processes that enable local and distant search. Because local search explores those potential solutions closest to initial ideas, it is best enabled by mind wandering. This is because mind wandering helps spread activation from initial ideas to its most closely related associates. Because distant search explores potential solutions that are further away from initial ideas, it is best enabled by mindful metacognition. This is because mindful metacognition helps redistribute activation from initial ideas to more remote ideas. This finding, demonstrated across two studies, has several implications for future research. Perhaps most intriguing among them is how even brief inductions of mindful metacognition can produce distant search, which has typically been conceptualized as resulting from failure in local search [10,11]. Our study design induced mindful metacognition in participants while they performed an ostensibly problem-unrelated task. It is worth exploring problem solving in individuals who bring a metacognitive stance to their problems in real-time, as opposed to invoking it after the fact as our participants did. A valuable complement to this finding could therefore study how dispositional levels of mindful metacognition relate to features of cognition implicated here such as the complexity of knowledge structures. Individuals who regularly redistribute activation away from initial ideas may have richer and more nuanced representations of their environment and the problems embedded in it.

Additionally, as our goal was to provide an initial test of these cognitive processes, our problems were rather simple relative to the more complex problems typically faced by individuals. It is possible that the effects of mindful metacognition will only increase with complex problems, as more rugged solution spaces have a greater number of peaks and valleys that limit local search. Time is another interesting topic for future research. Existing work suggests that the amount of time spent in the initial problem solving phase—but not the time spent on other tasks during breaks from problem solving—exerts an important influence on search [18]. We suggest that mindful metacognition could be increasingly relevant as time spent in initial problem solving increases, given its ability to help individuals bypass their initial ideas. Furthermore, given that individuals process their own initial ideas differently than they do ideas from external sources [28], it could be valuable to translate this body of work to group problem solving. In group problem solving, initial ideas proposed by one group member often impair search processes for other members [29]. Mindful metacognition could therefore be especially relevant in organizational settings, where a great deal of innovation occurs in groups [30].

In summary, the way we direct our cognitive processes has a direct impact on the type of solutions we have available to us. While getting lost in a stream of thoughts is most helpful when you are on the right track, for a fundamental change of perspective, our results suggest you may be best served by getting out of the stream and simply watching your thoughts go by.

## Supporting Information

S1 Dataset Search data for Study 1 and Study 2. (ZIP)
